# Visual outcomes of audio-luminous biofeedback training for a child with idiopathic nystagmus

**DOI:** 10.5935/0004-2749.20210026

**Published:** 2025-02-02

**Authors:** Monica Daibert-Nido, Yulia Pyatova, Michelle Markowitz, Samuel N. Markowitz

**Affiliations:** 1 Low Vision Service, (University Health Network Hospitals), Department of Ophthalmology and Vision Sciences, University of Toronto, Toronto, Ontario, Canada; 2 Private practice, Toronto, Ontario, Canada

**Keywords:** Nystagmus, pathologic/rehabilitation, Biofeedback, psychology, Low vision, Visual field tests, Nistagmo patológico/reabilitação, locus retiniano preferencial, Baixa visão, Testes de campo visual

## Abstract

Microperimetry biofeedback training is a vision rehabilitation method that involves the training of attention and oculomotor control, and the rehabilitation of poorly located and non-functional preferred retinal loci. It can significantly improve distance and near visual acuity in age-related macular degeneration. Previous studies have shown that biofeedback training using electrical nystagmography can reduce nystagmus amplitude and increase foveation time. However, these improvements have not been sustained following training sessions. We hereby report a pediatric case of idiopathic nystagmus in an 11-year old patient treated with microperimetric biofeedback to improve visual acuity and fixation stability. The training had a beneficial impact, positively affecting fixation stability as well as distance and near reading vision. Subjectively, improvement in quality of life was also reported. Conversely to previous studies, the positive effects in this case were maintained for as long as twelve months following therapy. To the best of our knowledge, this is the first case with long-term benefits to be reported in the literature.

## Abstract (pt)

O treinamento de *biofeedback* por microperimetria é um método de reabilitação da visão que envolve trei namento de atenção, controle oculomotor e reabilitação do *locus* preferencial de fixação da retina. Esse treinamento pode melhorar significativamente a acuidade visual para longe e perto na degeneração macular relacionada à idade. Estudos anteriores mostraram que o treinamento de *biofeedback* usando a nistagmografia elétrica pode reduzir a amplitude do nistagmo e aumentar o período de foveação. Entretanto, os resultados não se mantiveram após o término das sessões. Aqui é relatado um caso de tratamento com *biofeedback* por microperimetria para melhorar a acuidade visual e a estabilidade de fixação em uma criança de 11 anos de idade. O treinamento teve impacto benéfico e afetou positivamente a estabilidade da fixação e a visão para longe, para perto e de leitura. Subjetivamente, foi relatada melhoria da qualidade de vida. Em contraste com estudos anteriores, os efeitos positivos foram mantidos até 12 meses após a terapia. Até onde sabemos, este é o primeiro caso na literatura que relata benefícios de longo prazo.

## INTRODUCTION

Infantile idiopathic nystagmus syndrome (IINS) or congenital motor nystagmus is the most common type of infantile nystagmus. It is identified by a characteristic waveform eye movement pattern with an exponentially-increasing velocity of slow-phase followed by a saccadic fast phase. IIN is almost always bilateral and conjugate, and occurs in the horizontal plane in both upgaze and downgaze, with little variability^(1)^.

When the patient is asymptomatic, no treatment is required. However, if the patient’s visual acuity is decreased, or if they exhibit abnormal head posture, or oscillopsia, interventions are warranted. Traditional therapies include muscle surgery, optical devices, drugs, and botulinum toxin injections^(2,3)^.

Most current available therapies aim at changing the functional balance among eye muscles responsible for eye movements with the hope that they will improve ocular stability and foveation time for incoming images, resulting in better vision.

Active eye movement control training, an old intervention which is most common in low vision rehabilitation (LVR), has never been used in routine clinical practice in nystagmus cases with low vision for various reasons. One of these reasons is the inability to accurately document eye movements and fixation characteristics in patients with low vision, including nystagmus cases.

Biofeedback training (BT) is the latest technique for oculomotor control training in cases with low vision, and uses available modules of new microperimetry instruments, which can track and record eye movements characteristics^(4,5)^. The BT tracker system allows for real time audio and luminous BT, facilitating oculomotor control training through attentional techniques.

Previous studies using electrical nystagmogram have shown fixation stability improvement and enhancement of foveation time during audio BT, but only one of these cases reported using microperimetry^(6-8)^. No previous literature exists on the use of microperimetric BT for children with IINS. Thus, we report the long-term outcome of one successfully treated child with IINS to provide evidence for the feasibility of this new intervention in clinical pediatric nystagmus practice.

## CASE REPORT

The patient was first seen at the Low Vision Rehabilitation Service at the University of Toronto, and had previously been diagnosed with IIN, but no other pathologies. Informed consent was obtained from the parents.

During the first visit, data were collecting using Best Corrected Visual Acuity (BCVA) and distance vision with Early Treatment Diabetic Retinopathy Study (ETDRS) charts at 3 meters; as well as preferred retinal locus (PRL), fixation stability (FS), nystagmus amplitude estimates using the MAIA microperimeter (Centervue, Padua, Italy); and near vision testing (critical print size and reading acuity). Three months after the prescription and usage of regular glasses, prisms, and selective transmission lenses, the patient was offered BT.

The BT protocol involved four consecutive weekly sessions of training as described elsewhere^(5)^. Each session included four BT attempts of about 5-10 minutes each. The patient was given take-home efficiency reading exercises weekly, to be completed with the better eye near correction.

Endpoints included visual acuity for distance, fixation stability, nystagmus amplitude estimate and near reading acuity.

## RESULTS

**Figure 1:**
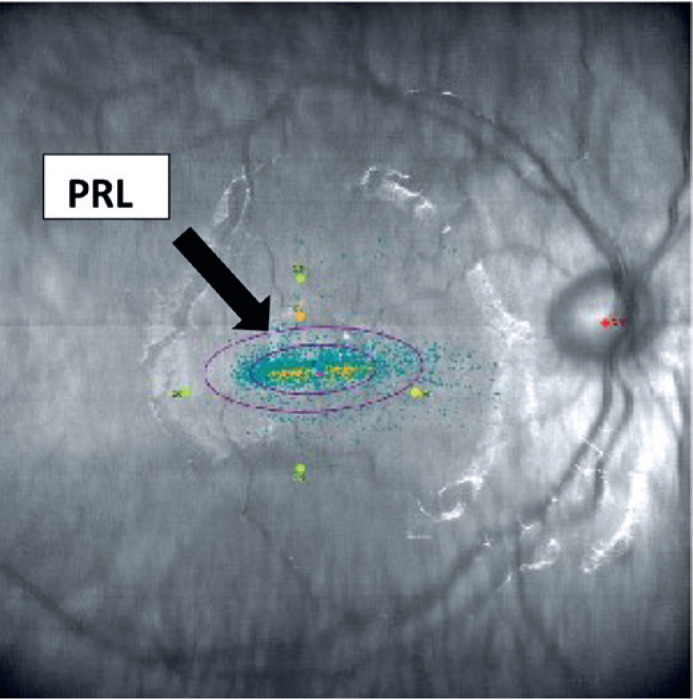
Bivariate contour ellipse area (BCEA) of the right eye (OD) pre biofeedback training.

The patient described in this report was an 11-year-old male with IINS. BCVA measured with ETDRS charts was 20/60-2 for the right eye (OD), 20/80 for the left eye (OS), and 20/50 with both eyes (OU). Better near vision was achieved with a +4.00 add that gave the patient 20/25 for near vision. Cycloplegic refraction was -5.50 sph + 3.50 cyl x 110 (OD) and -5.00 sph + 4.00 cyl x80 (OS). The patient was orthophoric, with a latent-manifest, jerk type, right-beating nystagmus with a null point in convergence. Five base-out prisms for each eye, a x 2.5 monocular telescope for distance spotting, and reading glasses with a +4.00 add were all prescribed. Tests using the MAIA microperimeter (Centervue, Padova, Italy), indicated a fixation stability of 13.1 squared degrees (sq; unstable fixation) and a nystagmus amplitude estimate of 11.3 degrees in the OD (Figure 1). The OD eye was selected for BT.

**Figure 2:**
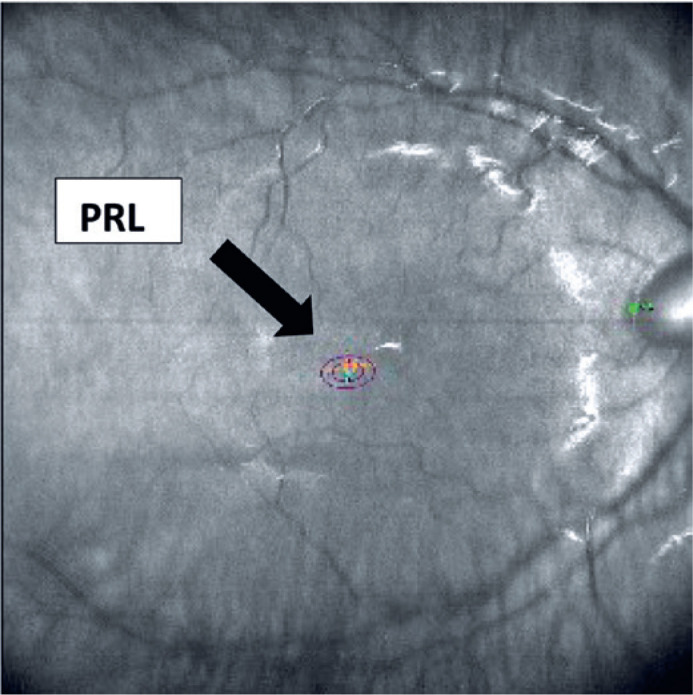
Bivariate contour ellipse area (BCEA) of the right eye (OD) 3 months post biofeedback training.

The foveola was marked as the trained retinal locus target. Three months following BT, BCVA improved from 20/60 to 20/30 in the OD, from 20/80 to 20/32 in the OS, and from 20/50 to 20/32 in the OU (Table 1). The patient’s near vision improved from 20/25 with a +4.00 add to 20/20 without the use of an add. Fixation stability improved from 13.1 to 1.2 sq in the right eye. The nystagmus amplitude estimate improved from 13.1 to 2.5 degrees (Figure 2). Subjectively, the patient reported an improvement in distance and reading at school and while playing video games. Twelve months after BT, the visual acuity measured in the OD was 20/40, in the OS was 20/40, and with both eyes was 20/32. The fixation stability was 3.5 sq.

**Table 1:** Summary visits at baseline, 1-4 months, and 3 months follow-up

Visit	VA logmar OD	VA logmar OS	VA logmar OU	BCEA Ave. (sq) OD	Amplitude Ave. (deg) OD
Baseline	0.49	0.60	0.39	13.1	11.3
V1	0.30	0.60	0.30	5.9	11.4
V2	0.30	0.30	0.20	4.9	9.8
V3	0.30	0.30	0.20	5.7	9.7
V4	0.30	0.30	0.20	3.5	9.1
3 mo. follow-up	0.20	0.30	0.20	1.2	2.5
12 mo. follow-up	0.30	0.30	0.20	3.5	

VA= visual acuity (LogMAR ETDRS); OD = right eye; OS= left eye; OU= both eyes; BCEA= bivariate contour ellipse area; Ave= average; sq= squared degrees; deg= degrees; V= visit; mo= month.

## DISCUSSION

The use of BT in nystagmus was described as early as 1980^(6)^. Yet, the utilization of this rehabilitation method never reached the clinical environment for various reasons. Mostly, this was because the required instrumentation remained as laboratory-based devices which tested various aspects of the proposed training. In addition, no methodology was developed to follow the long-term efficiency of the method, resulting in persistent doubts regarding its efficiency^(7)^. Recently, following the introduction of microperimetry instruments^(5)^ in the clinical environment, BT was revisited for the treatment of nystagmus, and positive outcomes were published in the literature^(8)^. According to these reports, it seems that the core of the BT method is based on improving oculomotor control through attention, yet its mechanism of action is still not completely understood^(7,9)^.

Our study comprised of one clinical nystagmus case which was treated with BT, with extremely positive results. As reported above, a significant improvement in distance visual acuity was seen, which was confirmed by a corresponding reduction in fixation stability, a significant reduction in nystagmus amplitude estimates, and a report of improved general quality of life parameters. In addition, the effects of the training were maintained one year following the end of the training, which has never been reported in the literature previously.

While previous studies used electrical nystagmography connected to audio BT, our case was trained using microperimetry with audio-luminous BT. This difference in methodology may explain the more positive outcomes found in our study in comparison to previous studies. These previous studies did not report a sustained benefit of BT, while our case was able to maintain this benefit for one year. The younger age of our patient, which may have been related to increased brain plasticity in the patient^(10)^, possibly contributed to this difference.

It is feasible for the BT protocol used in this study to be applied in a clinical setting. This study has demonstrated very promising outcome measures following BT, thus promoting greater experimentation with this rehabilitation method in clinical practice. A prospective randomized trial is required in the future to verify the results of this study.
